# From Biomass-Derived *p*-Hydroxycinnamic Acids to Novel Sustainable and Non-Toxic Phenolics-Based UV-Filters: A Multidisciplinary Journey

**DOI:** 10.3389/fchem.2022.886367

**Published:** 2022-07-05

**Authors:** Benjamin Rioux, Jeanne Combes, Jack M. Woolley, Natércia d. N. Rodrigues, Matthieu M. Mention, Vasilios G. Stavros, Florent Allais

**Affiliations:** ^1^ URD Agro-Biotechnologies Industrielles (ABI), CEBB, AgroParisTech, Pomacle, France; ^2^ Department of Chemistry, University of Warwick, Coventry, United Kingtom; ^3^ Lipotec SAU, Barcelona, Spain

**Keywords:** *p*-hydroxycinnamic acids, synthetic biology, biotechnology, in stream product recovery, green chemistry, Knoevenagel, UV-filter, photodynamics

## Abstract

Although organic UV-filters are extensively used in cosmetics to protect consumers from the deleterious effects of solar UV radiation-exposure, they suffer from some major drawbacks such as their fossil origin and their toxicity to both humans and the environment. Thus, finding sustainable and non-toxic UV-filters is becoming a topic of great interest for the cosmetic industry. A few years ago, sinapoyl malate was shown to be a powerful naturally occurring UV-filter. Building on these findings, we decided to design and optimize an entire value chain that goes from biomass to innovative biobased and non-toxic lignin-derived UV-filters. This multidisciplinary approach relies on: 1) The production of phenolic synthons using either metabolite extraction from biomass or their bioproduction through synthetic biology/fermentation/in stream product recovery; 2) their functionalization using green chemistry to access sinapoyl malate and analogues; 3) the study of their UV-filtering activity, their photostability, their biological properties; and 4) their photodynamics. This mini-review aims at demonstrating that combining biotechnology, green chemistry, downstream process and photochemistry is a powerful approach to transform biomass and, in particular lignins, into high value-added innovative UV-filters.

## 1 Introduction

Faced with the damage accrued to coral reefs which has been linked to toxic UV-filters, such as oxybenzone and octinoxate, many territories, including Hawaii in 2018, have banned the use of sunscreen lotions containing such UV-filters (Ouchene et al., 2019). Besides being harmful to the Environment, these chemicals are also suspected to be hazardous to Humans, with potential toxicities toward thyroid, testosterone level, kidney function and pubertal timing (Suh et al., 2020). Time is therefore ripe to find non-toxic alternatives to these UV-filters. One strategy to tackle this challenge consists in mimicking photoprotection molecules derived from Nature (i.e., “nature inspired”); specifically, UV-filters involved in plant-defense mechanisms. Indeed, Zwier and co-workers, demonstrated that sinapoyl malate, a plant metabolite, exhibited potent UV-filtering activity (Dean et al., 2014). Targeting non-toxic UV-filters has considerable potential, and thus synthesizing these *via* a sustainable synthetic route could be transformative to the skincare industry. Indeed, although octinoxate could be bio-sourced from *p*-coumaric acid (one of the 4 main *p*-hydroxycinnamic acids found in Nature), it is entirely petrochemical-based and its synthesis involves fossil fuel-reagents, meaning it is far from being considered as a green process with regards to the 12 principles of Green Chemistry (Anastas and Warner, 1998).

Building on these findings, we decided to design sustainable synthetic pathways toward novel non-toxic sinapoyl malate analogues, starting from biomass-derived building blocks (i.e., 2nd generation sugars, *p*-hydroxycinnamic acids and corresponding benzaldehydes). This ambitious project, that aims at designing and optimizing a value chain that goes from biomass to innovative bio-based UV-filters, relies on a multidisciplinary approach that combines expertise in synthetic biology, fermentation, downstream process, green chemistry and photochemistry. This mini-review describes, in sequence, the different stages of this value chain: 1) microorganism metabolic engineering and *in situ* product recovery for the bioproduction of *p*-hydroxycinnamic acids and corresponding *p*-hydroxybenzaldehydes, 2) the design of innovative sustainable synthetic routes toward new nature inspired UV-filter starting from the *p*-hydroxybenzaldehydes, 3) the assessment of the biological properties of these UV-filters, as well as 4) their physico-chemical properties. It is worth mentioning that, to the best of our knowledge, such an integrated approach has never been reported in the literature. Therefore we specifically focus on our own groups’ work but draw reference, where appropriate, to complementary studies by other groups.

## 2 Discussion

### 2.1 Bioproduction of *p*-Hydroxycinnamic Acids and Corresponding *p*-hydroxybenzaldehydes

#### 2.1.1 Heterologous Bioproduction of *p*-Hydroxycinnamic Acids

As microorganisms are able to produce *de novo* aromatic amino acids (AAAs)—i.e., L-Tyrosine (Tyr) and L-Phenylalanine (Phe)- through the shikimate pathway, they are a promising heterologous producer of derived molecules. Hence, a great deal of research has been carried out on this topic, as illustrated by the number of recent reviews from leading researchers in the field (Suástegui and Shao, 2016; Huccetogullari et al., 2019; Averesch and Kayser, 2020; Cao et al., 2020; Liu et al., 2020; Shen et al., 2020; Dickey et al., 2021).

**FIGURE 1:**
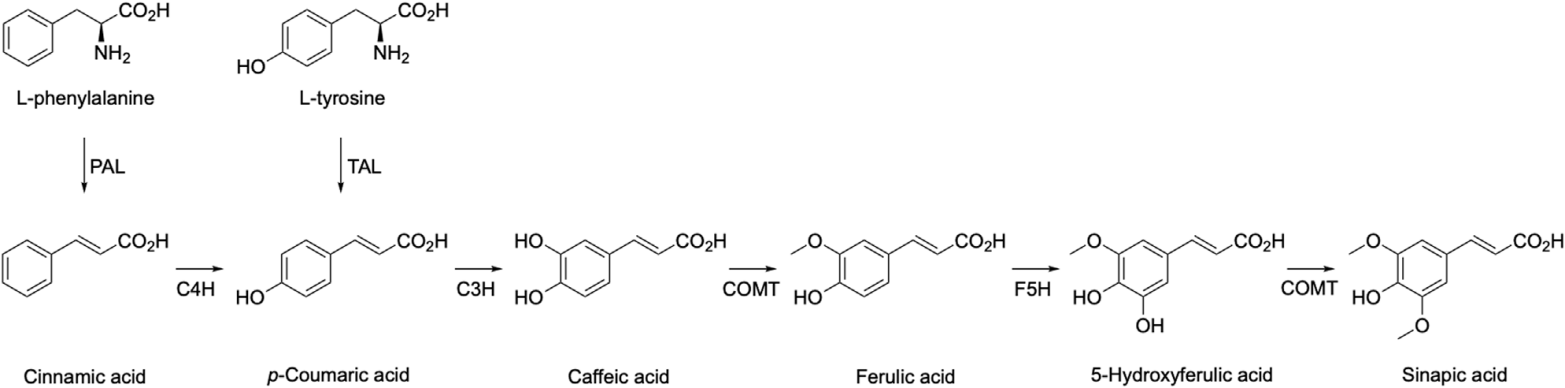
*p*-Hydroxycinnamic acids biosynthesis pathway. (PAL: phenylalanine ammonia-lyase, TAL: tyrosine ammonia-lyase, C4H: cinnamate-4-hydroxylase, C3H: *p*-coumarate-3-hydroxylase, COMT: caffeic acid *O*-methyl transferase, F5H: ferulic-5-hydroxylase).

*p*-Hydroxycinnamic acids are AAA derived molecules and, therefore, can be produced *de novo* through the heterologous expression of the relevant phenylpropanoid pathway enzymes presented Figure 1 (Vargas-Tah and Gosset, 2015). Chemical synthesis or extraction from plant biomass are two additional routes to access these *p*-hydroxycinnamic acids; however, they have drawbacks that biotechnological routes do not have. A recent review by Allais and co-workers discusses these three routes, with specific focus on the promising aspects of the biotechnology route (Flourat et al., 2021).

The latter heterologous biosynthesis using microorganisms starts with the deamination of Tyr and Phe by ammonia lyases to produce, respectively, *p*-coumaric acid (*p*-CA) and cinnamic acid. Cinnamic acid can then be hydroxylated into *p*-coumaric acid by a cinnamic acid 4-hydroxylase. *p*-CA is the first *p*-hydroxycinnamic acid of the pathway and it has been heterologously produced at industrially relevant titers. In fact, DuPont produced significant amount of *p*-CA from D-glucose in a two-step patented process using two engineered *Escherichia coli* strains with the final aim of producing 4-vinylphenol (Ben-Bassat et al., 2005; Sariaslani, 2007). Moreover, Liu *et al.* in 2019 reached a final production of 12.5 g.L^−1^ of *p*-CA from D-glucose in a fed-batch fermentation using an engineered *Saccharomyces cerevisiae* strain. Although *p*-CA production has been reported in various other promising microorganisms including *Pichia pastoris* (Chen et al., 2021), *Pseudomonas putida* (Nijkamp et al., 2007; Calero et al., 2016), (Nijkamp et al., 2007; Calero et al., 2016), a *S. cerevisiae* that can use xylose as a sole carbon source (Borja et al., 2019) and *Yarrowia lipolytica* (Gu et al., 2020), the productions did not reach an industrially attractive titer yet and need further development.

The expression of a *p*-CA 3-hydroxylase allows the formation of caffeic acid from *p*-CA. The highest *de novo* bioproductions are, once again, with engineered *E. coli* and *S. cerevisiae* strains: 766.7 mg L^−1^ and 569.0 mg L^−1^ of caffeic acid, respectively (Huang et al., 2013; Zhou et al., 2021).

Following this, a caffeic acid *O*-methyltransferase allows the bioconversion of caffeic acid into ferulic acid. Kang et al., in 2012, (Kang et al., 2012), obtained the highest *de novo* ferulic acid biosynthesis published to-date: 196 mg L^−1^ from D-glucose with an engineered *E. coli*. Since this finding, most of the work focuses on producing *de novo* ferulic acid-derived molecules.

To the best of our knowledge, no work has been published yet on *de novo* heterologous production of 5-hydroxyferulic acid and sinapic acid in any microorganism.

**FIGURE 2:**
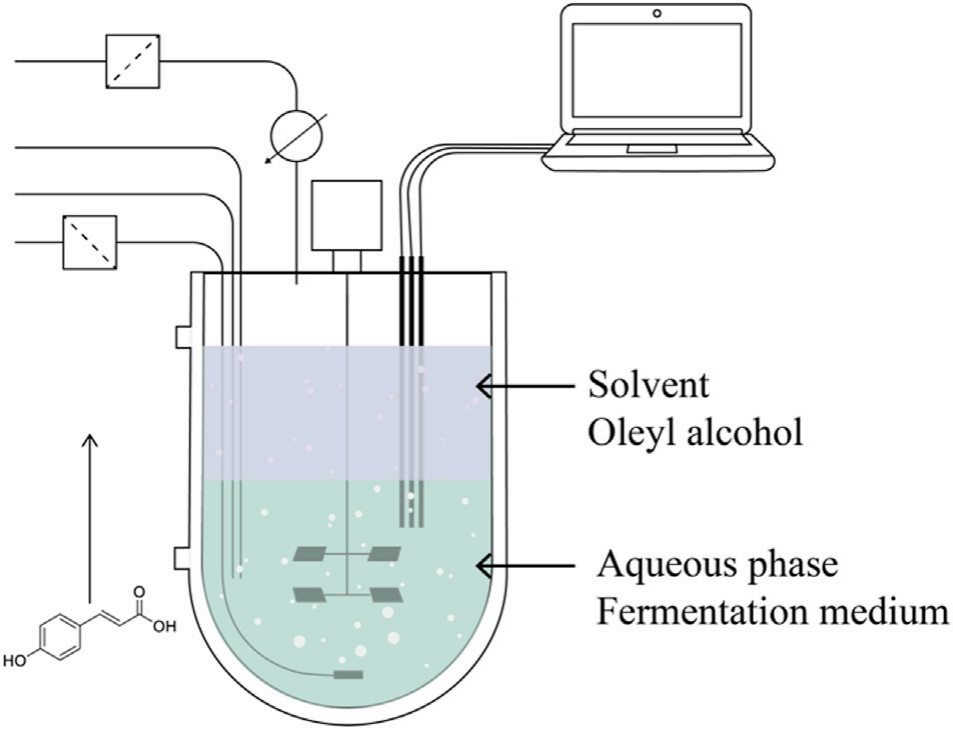
Continous recovery through liquid-liquid extraction (from Combes et al., 2021).

To summarise, most of the work concerning *de novo* bioproduction of *p*-hydroxycinnamic acids focuses on rewiring carbon flux towards Tyr and Phe synthesis through metabolism engineering, deletion of competing pathways, relief of feedback inhibitions, overexpression of rate-limiting enzymes and heterologous expressions of the relevant genes. However, some limitations are not straightforward to address with such strategies. Indeed, since hydroxycinnamic acids are hydrophobic compounds, their solubility in aqueous solutions such as fermentation media is low (Mota et al., 2008), they are also known to be antimicrobial agents (Alves et al., 2013; Pernin et al., 2019), hence, their accumulation in the broth leads to toxicity towards the producer microorganism (inhibition). In a previous study, a different strategy was proposed to simultaneously address all these concerns, by focusing on the intensification through process optimization and, more precisely, on the implementation of an *in situ* product recovery (ISPR) (Combes et al., 2021). The continuous recovery of heterologously synthesized *p*-CA through liquid-liquid extraction was implemented (Figure 2), enabling the detoxification of the medium and the enhancement of accumulation limits. This ISPR process coupled to fermentation is called biphasic fermentation, and in this particular case, oleyl alcohol was used as the extractant solvent.

Within this work, an endogenous decarboxylation of *p*-CA into 4-vinylphenol from the engineered *S. cerevisiae* strain was highlighted. The continuous removal of *p*-CA prevented this competitive pathway as well, intensifying further *p*-CA *de novo* production. This work demonstrates the importance and intensification capacities of process optimization for the heterologous microbial production of *p*-hydroxycinnamic acids. This approach will help engineer a viable biotechnological process, as it also eases the separation steps.

Through strain engineering using the many tools and strategies of molecular biology and using process engineering and intensification, the heterologous bioproduction of *p*-hydroxycinnamic acids appears to be a promising, efficient and sustainable route (Krömer et al., 2020). *p*-Hydroxycinnamic acids are platform molecules and notably, allow easy access to the corresponding *p*-hydroxybenzaldehydes as detailed in the next section.

#### 2.1.2 Biotransformation of *p*-hydroxybenzaldehydes From *p*-Hydroxycinnamic Acids

Vanillin (4-hydroxy-3-methoxybenzaldehyde), the most important flavoring agent in the industry, is a *p*-hydroxybenzaldehyde derived from ferulic acid. The significance and market size of vanillin, and especially natural vanillin, has led to very active research towards its biotechnological production, as extraction from plant biomass could not meet the market demand (Priefert et al., 2001). One popular strategy to biotechnologically produce natural vanillin is to use ferulic acid as substrate of the biotransformation. Some microorganisms are able to metabolize ferulic acid, and vanilla is one of the degradation intermediates. Indeed, since 2000, Rhodia (now Solvay) uses a wild *Streptomyces setonii* strain to bioconvert Rhovanil Natural (natural vanillin) from ferulic acid. The latter was obtained through a technology developed by Givaudan comprising the metabolism of ferulic acid by the strain, and the separation of the different products including vanilla, up to 16 g L^−1^ (Muheim et al., 1998). Although the main strategy consists in using strains able to metabolize ferulic acid into vanillin, other strategies exist, including: 1) Genetically engineering those strains to increase their capacities; 2) heterologously expressing the enzymes of interest in other strains; and 3) producing *de novo* vanillin, combining previous detailed pathway (Section 2.1) and the enzymes enabling the biotransformation of ferulic acid into vanillin (Martău et al., 2021). Many reviews cover published strategies of successful ferulic acid biotransformations into vanillin and we refer the reader to these for further information (Priefert et al., 2001; Walton et al., 2003; Gallage and Møller, 2015; Martău et al., 2021).

For other corresponding *p*-hydroxybenzaldehydes, there is less published work, due to smaller industrial interest. Yet, there is evidence that the aforementioned strategy works for *p*-CA and sinapic acid biotransformation into their corresponding *p*-hydroxybenzaldehyde form, *p*-hydroxybenzaldehyde and syringaldehyde, respectively (Estrada Alvarado et al., 2001; Nimura et al., 2010).

Unfortunately, there are two major bottlenecks for this biotechnological approach: The formation of undesired by-products (e.g., oxidation of *p*-hydroxybenzaldehydes), often overcome by deletion of the enzymes of interest, and the toxicity of the *p*-hydroxybenzaldehyde. The latter has been overcome first by choosing tolerant strains, but also by the implementation of an in-stream product recovery process to the biotransformation (Hua et al., 2007; Sciubba et al., 2009). Such implementation could also resolve the issue related to competitive metabolic pathway as previously described for the case of *p*-CA decarboxylation and the biphasic fermentation study (Section 2.1).

To summarize, in one-step or two-step biotechnological approaches, *p*-hydroxybenzaldehydes can be produced from glucose, and it is well established for the ferulic acid-vanillin route. For the other *p*-hydroxycinnamic acids, there is still much work to be done to create viable routes, as they remain at the proof-of-concept stage. In other words, these biotechnological processes open the way for the production of novel UV-filters from simple sugar using engineered microorganisms.

Now that the bioproduction of *p*-hydroxybenzaldehydes has been addressed, we move to discuss the synthesis of UV-filters from these biotechnologically-generated phenolics building blocks.

### 2.2 Synthesis of UV Bio-Based Filters From Corresponding *p*-hydroxybenzaldehydes

**SCHEME 1:**
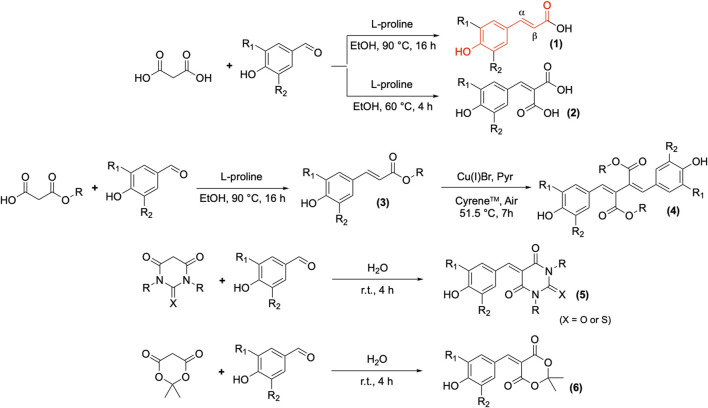
Synthesis of *p*-hydroxycinnamic acids **(1)** and their derivatives: *p*-hydroxycinnamic diacids **(2)**, *p*-hydroxycinnamic esters **(3)**, *β*-*β* dimers **(4)**, *p*-hydroxycinnamyl barbiturics **(5)**, and *p*-hydroxycinnamyl Meldrum’s **(6)** (R_1_ and R_2_ = H, OH or OMe).

*p*-Hydroxycinnamic acids are available in a variety of common vegetables, especially in *Brassicaceae* (e.g., mustard, rapeseed, kale) (Cartea et al., 2011). Extraction of phenolic compounds from those vegetables by-products would be of great interest to obtain natural alternatives to synthetic molecules. However, their extraction remains a challenge as those phenolic compounds are sensitive to drastic conditions of high-temperature, oxygen and pH (Charlton and Lee, 1997). Several methods of extraction are described in the literature (Esclapez et al., 2011; Galanakis et al., 2013; Flórez et al., 2015), but the phenolic compounds recovered remains in low concentration, requiring further purification steps and leading to process which are not yet economically viable at industrial scale (Xu and Diosady, 2002; Prapakornwiriya and Diosady, 2014). Therefore, the synthesis of *p*-hydroxycinnamic derivatives, with regards to the 12 principles of green chemistry, remains the primary pathway to obtain new phenolic UV-filters. The main synthetic route to access *p*-hydroxycinnamic acids in high yield and large scale consists in the condensation of malonic acid with *p*-hydroxybenzaldehydes through the Knoevenagel–Doebner reaction, which has been known for decades. Originally, this reaction involved using a large amount of pyridine as solvent, and amine as catalyst [i.e., aniline or piperidine (Knoevenagel, 1894)]. To enhance yields, reduce reaction time, and limit/avoid the use of a toxic solvent (Pollock et al., 1943) or catalyst, several optimizations were carried out in the presence of alternative solvents and catalysts (e.g., DMSO (Hedge et al., 1961), DMF (Shi et al., 2000), ionic liquids (Forbes et al., 2006; Hu et al., 2016), water/ethanol with cobalt ferrite nanoparticles (Rajput and Kaur, 2013), water with 3-aminopropylated silica gel (Isobe et al., 2005), ammonium salts (van Schijndel et al., 2017) or L-tyrosine (Thirupathi et al., 2012)] or activated under microwave to drastically shortened the reaction time (Singh and Kaur, 2011; Mouterde and Allais, 2018). More recently, sustainable Knoevenagel condensation procedures, based on green chemistry principles (Anastas and Warner, 1998), have been applied to synthesize *p*-hydroxycinnamic acids. For instance, pyridine and aniline were substituted by ethanol and L-proline as solvent and catalyst, respectively, both of which are safe for human health and eco-friendly (Peyrot et al., 2019; Rioux et al., 2020). This provided access to natural *p*-hydroxycinnamic acids in high yield and at large scale with a green and sustainable synthesis. Moreover, these *p*-hydroxycinnamic acids exhibited very interesting UV-filtering properties for both the UV-B (280–315 nm) and the UV-A (315–400 nm) regions of the electromagnetic spectrum and are known for their potential to act as UV-filters or boosters (*i.e.*, to enhance the photoprotective properties of anti-UV formulations) (Peres et al., 2018). For example, *p*-hydroxycinnamic acids (sinapic, ferulic, caffeic, and coumaric) were used in formulation and proved comparable or better than commercial filters (Peres et al., 2018; Surendran et al., 2019; Sauce et al., 2021). Such UV-filtering properties derive from the conjugated backbone (shown in red) of these natural compounds (Scheme 1) and the steric hinderance applied on the *β* position of the C=C double bond, which are essential for good absorption and photostability upon UV exposure. On the other hand, substituents *R*_*1-2*_, as well as the carboxylic function and the *β* position, can be readily modulated to access the corresponding esters, thus allowing to fine tune the properties of those molecules.

The aforementioned green Knoevenagel-Doebner condensation procedures unlocked a quick and easy access to natural *p*-hydroxycinnamic acid derivatives **(3)** (Scheme 1), such as sinapoyl-L-malate (Peyrot et al., 2020c), identified in the leaf to be responsible for photoprotection, or sinapine (Mouterde et al., 2020), mainly accumulated in roots, the two most common esters of sinapic acid in plants (Nićiforović and Abramovič, 2014).

**FIGURE 3:**
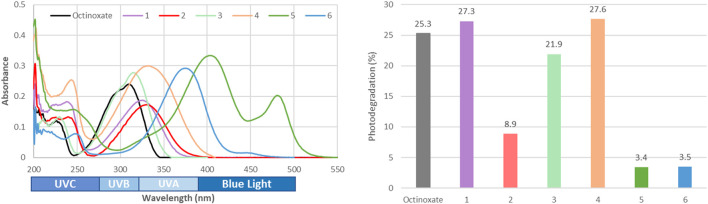
UV-Vis spectra in EtOH (C = 10 µM) of each series of p-hydroxycinnamic derivatives with octinoxate as reference and their respective loss of absorbance upon 1 h of UV radiation (λ = 300 nm, *p* = 8.32 W/m^2^, stirring, *T* = 35°C).

Based on those natural structures, several modifications were implemented around the phenol and the *β* position in order to further modulate the UV-filtering properties (Figure 3). The presence of a free phenol provided opportunity to perform biomimetic radical-mediated reactions in green solvent (i.e., Cyrene®) leading to *β*-*β* dimers of sinapoyl esters **(4)** (Scheme 1) (Mention et al., 2020). Such molecules turned out to provide a full coverage of the UV-A and UV-B regions of the electromagnetic spectrum, by increasing the conjugation throughout a longer backbone. It is noteworthy to mention that the free phenol can also be functionalized in order to be grafted onto materials—providing them with anti-UV properties (Peyrot et al., 2020a; Joram Mendoza et al., 2020; Mendoza et al., 2021a; Mendoza et al., 2021b)—or even be used for polymerization to directly provide anti-UV materials (Sasiwilaskorn et al., 2008). As previously mentioned, an increased steric hinderance on the *β* position is a key factor to improve absorption and stability against UV radiation exposure. One way to easily introduce a substituent on the *β* position is to perform a classic Knoevenagel condensation (i.e., no decarboxylation step) to offer a second carboxyl group **(2)** (Rioux et al., 2020) that can be functionalized further by (trans)esterification (Horbury et al., 2019). The compounds resulting from this modification exhibited drastically improved stability toward UV radiation exposure, while retaining their initial wavelength coverage and level of absorption (Figure 3), making them promising, nature-inspired UV-filters. Furthermore, some strong Brønsted acids, such as Meldrum’s acid or (thio)barbituric acid can also be used to synthesize highly hindered compounds (Peyrot et al., 2020b; Abiola et al., 2021a; Abiola et al., 2021b; Rioux et al., 2022). The high acidity of their hydrogen at the β positions of the two carbonyls allows the implementation of base-free synthetic procedures perfectly fitting green chemistry by using water as solvent and catalyst at room temperature and without the need of purification, as *p*-hydroxycinnamyl (thio)barbiturics **(5)** and Meldrum’s **(6)** readily precipitate in water (Scheme 1). The symmetric structures of (**5**) and (**6**) lead to a bathochromic shift in absorbance (380–500 nm), as well as covering both the UV-A and UV-B (Figure 3). Naturally occurring phenols (i.e., *p*-hydroxybenzaldehydes and *p*-hydroxycinnamic acids) proved to be promising starting materials to obtain compounds of interest as replacements for the current petroleum-based filters used in cosmetics, exhibiting favourable properties—which can be readily modulated through precise modification of their structure—as UV-filters.

Having discussed the synthesis of *p*-hydroxycinnamic acid derivatives through green procedures and discussed their UV-filtering capacity, we now move to evaluate their biological properties, with specific focus on antioxidant activity, tyrosinase inhibition and endocrine disruption.

### 2.3 Biological Properties of the Novel UV Bio-Based Filters

UV-filters are of interest in a wide range of applications either to directly protect the consumer against UV radiation exposure or to prevent degradation of goods caused by exposure to UV radiation. In recent years, several molecules widely used in sunscreen formulations have been subject to criticism, mainly due to their potential toxicity towards human (Matta et al., 2019; Matta et al., 2020) and environmental health (Schneider and Lim, 2019). As a consequence, the need to provide safe, bio-based and eco-friendly alternatives has grown exponentially. One way to restrain the risks is to limit the number of compounds used in the formulation with multifunctional molecules that can cover different facets at the same time (e.g., UV-filter, antioxidant) while retaining biological properties of more complex formulations.

#### 2.3.1 Antioxidant Activity

**FIGURE 4:**
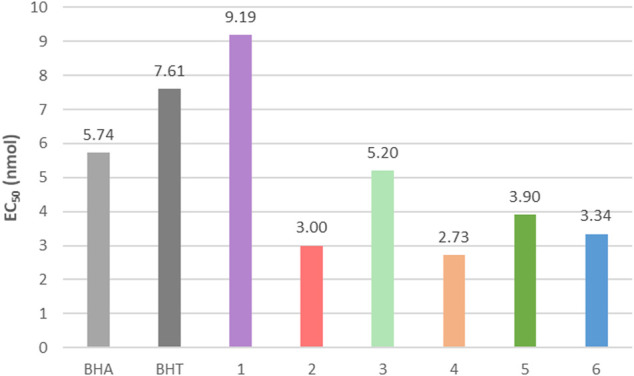
Examples of EC_50_ for each series of *p*-hydroxycinnamic acids derivatives relating to free radical inhibition in ethanol, compared to antioxidants conventionally used: BHA and BHT.

Antioxidants are essential to prevent damage from UV radiation exposure by neutralizing the Reactive Oxygen Species (ROS) that may form (Krutmann, 2003; Liebel et al., 2012). ROS, in the form of free radicals, are highly reactive species capable of degrading materials and inducing cellular damage in living organisms (i.e., inflammation, oxidative stress or even carcinogenesis) (Pillai et al., 2005; Austin et al., 2018). Phenols, especially the aforementioned *p*-hydroxycinnamic acids and their derivatives, are known to exhibit interesting antioxidant activity through their high conjugation, facilitating their ability to neutralize free radicals (Peyrot et al., 2020a; Peyrot et al., 2020c; Mention et al., 2020; Mouterde et al., 2020; Rioux et al., 2020; Abiola et al., 2021a; Abiola et al., 2021b). Such molecules have proven to be competitive (Figure 4, notably those derived from caffeic, ferulic and sinapic acid), against the antioxidants conventionally used and that are strongly criticized for their suspected endocrine toxicity and carcinogenic effect like BHA (butylated hydroxyanisole), BHT (butylated hydroxytoluene) or Trolox (6-hydroxy-2,5,7,8-tetramethylchroman-2-carboxylic acid). With their ability to scavenge ROS, paired with a capacity to absorb UV radiation, these *p*-hydroxycinnamic acids derivatives are compounds of choice to protect from UV radiation exposure and its consequences.

#### 2.3.2 Tyrosinase Inhibition Activity

**FIGURE 5:**
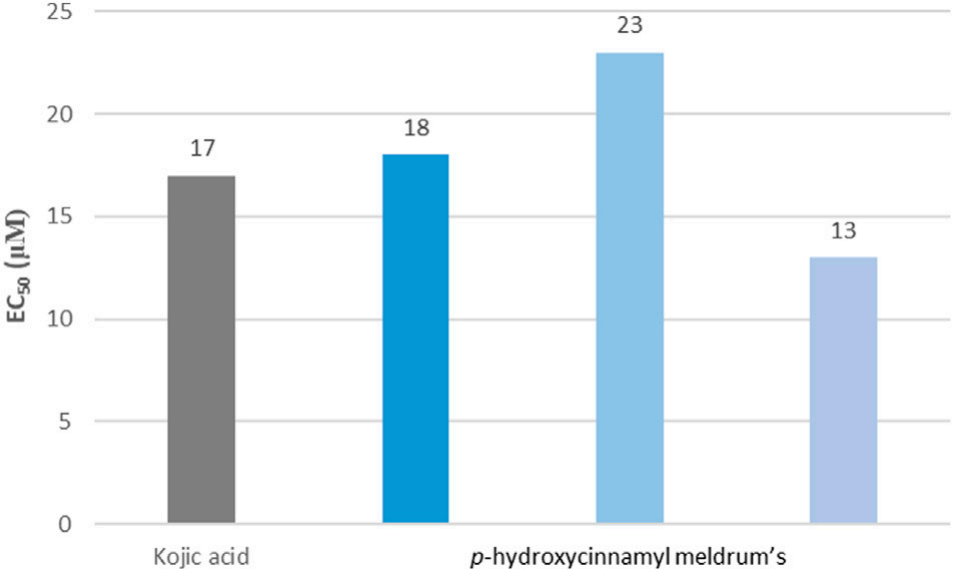
Examples of EC_50_ for the *p*-hydroxycinnamyl Meldrum’s series compared to Kojic acid for mushroom tyrosinase inhibition.

Excessive exposure to the sun can also induce hyperpigmentation in the form of age spots on the exposed skin (Vashi and Kundu, 2013). This negative effect caused by cell degeneration is due to an abnormal production of melanin, a pigment usually produced to tan the skin and protect it against UV radiation exposure. One way to reduce the effect of this disorder is to inhibit tyrosinase activity, responsible for the production of melanin during melanogenesis. Tyrosinase inhibition, widely described in the literature, can be carried out using fungal tyrosinase to mimic human tyrosinase, allowing the *in vitro* identification of potential inhibitors (Chakraborty et al., 1998; Chawla et al., 2008; Minsat et al., 2021). By using Kojic acid as a reference (Neeley et al., 2009; Minsat et al., 2021), some compounds proved to have great potential as tyrosinase inhibitors, in particular *p*-hydroxycinnamyl Meldrum’s **(6)** (Figure 5) (Peyrot et al., 2020b). This secondary activity, paired with their UV-filter activity and antioxidant properties, allows *p*-hydroxycinnamic acid derivatives to offer further protection against an over exposition to the sun.

#### 2.3.3 Endocrine Disruption Activity

**FIGURE 6:**
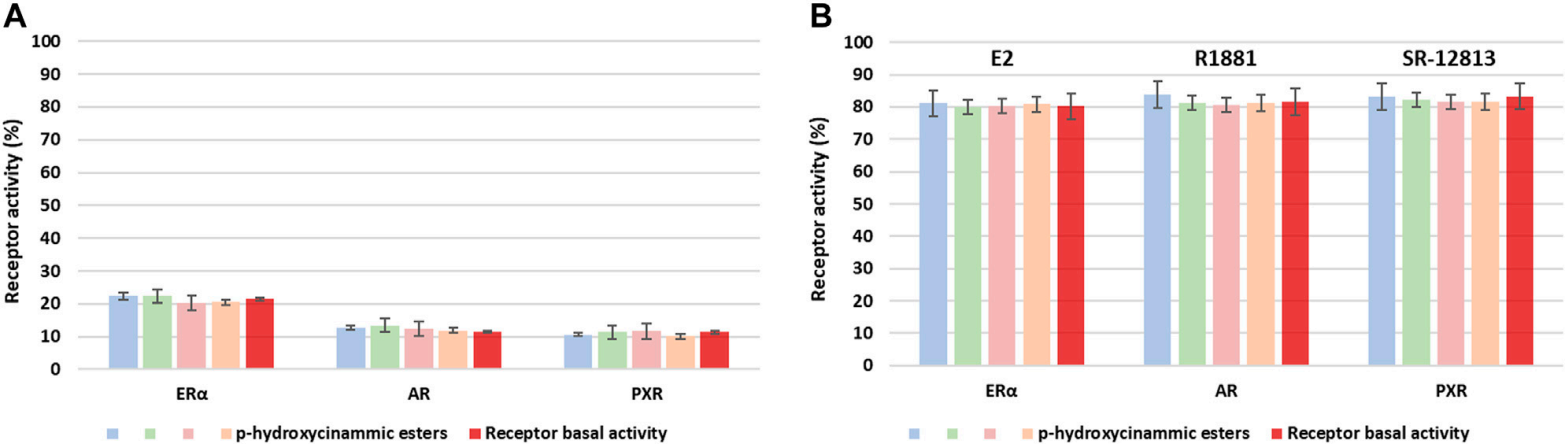
Receptor activity (%) of estrogen receptor *α* (ER*α*), androgen receptor (AR) and pregnane X receptor (PXR) concerning agonist **(A)** and antagonist **(B)** interactions of *p*-hydroxycinnamic esters at 10 μM.

As mentioned in the introduction, petroleum-based molecules currently used in formulations are criticized for their potential toxicity, both on humans and the environment. One of the main criteria required for the novel bio-based alternatives to efficiently replace them is to ensure their innocuous nature. Recently, some studies have highlighted the ability of organic UV-filters to be endocrine disruptors (Maipas and Nicolopoulou-Stamati, 2015), which can cause serious negative effects on the central nervous system and reproductive organs (Crews and McLachlan, 2006). As a preliminary step to determine potential toxicity, the interactions between several aforementioned *p*-hydroxycinnamic acid derivatives and endocrine receptors were evaluated *in vitro* (Horbury et al., 2019; Peyrot et al., 2020b; Peyrot et al., 2020c; Abiola et al., 2021b). Regardless of the modification introduced on the molecules, no agonist or antagonist interactions toward the different receptors were found (Figure 6), as they kept their normal activity even while in the presence of high concentration of the studied molecules. With the innocuousness determined by this preliminary analysis, combined with the versatile properties of *p*-hydroxycinnamic acid derivatives toward the protection against UV radiation exposure, those compounds can be considered as serious bio-based alternatives of current organic UV-filters.

After having addressed the bioproduction of *p*-hydroxybenzaldehydes, their use for the synthesis of new nature-inspired UV-filters, the assessment of their UV-filtering and biological properties as well as endocrine toxicity, we conclude this review by considering how these nature-inspired UV-filters deal with radiation exposure at the molecular level.

### 2.4 Physico-Chemical Properties of the UV Bio-Based Filters

The main photophysical requirement for an ideal UV-filter is to strongly absorb UV radiation. However, the absorption of UV radiation promotes molecules onto high energy excited states, and this excess energy must necessarily be dissipated *via* a combination of photophysical and photochemical processes (Baker and Stavros, 2016; Rodrigues and Stavros, 2018; Holt and Stavros, 2019; Abiola et al., 2020; Bacardit and Cartoixà, 2020). Photophysical processes relate (in part) to intramolecular energy transfer, while photochemical processes imply the breaking or making of chemical bonds, such as the generation of a photoproduct. The combination of these processes is referred to as the molecule’s “photodynamics,” and it ultimately defines the behavior and efficiency of a UV-filter.

Ideal UV-filters should dissipate excess energy as harmless heat, without compromising their structural integrity, generating any reactive species, or otherwise prompting harmful side chemistry processes (Baker and Stavros, 2016; Baker et al., 2017; Rodrigues and Stavros, 2018; Holt and Stavros, 2019; Abiola et al., 2020). This ideal behavior is typically facilitated by Internal Conversion (IC) i.e., non-radiative transition between energy levels of a given molecule (Baker and Stavros, 2016; Holt and Stavros, 2019). Similar to other photophysical processes, IC takes place on a femtosecond (10^–15^ s, fs) to picosecond (10^–12^ s, ps) timescale, hence the study of UV-filter photodynamics requires the use of ultrafast laser spectroscopy techniques (Abiola et al., 2020; Venkatraman and Orr-Ewing, 2021). In particular, transient absorption spectroscopy (electronic and vibrational) experiments have proven extremely useful in unveiling the ultrafast photodynamics of UV-filters (Baker and Stavros, 2016; Rodrigues and Stavros, 2018; Holt and Stavros, 2019; Abiola et al., 2020). The photodynamics of UV-filters affect their macroscopic properties, and it is therefore common for ultrafast laser spectroscopy studies to be complemented with steady-state experiments which, for example, evaluate changes in absorbance before and after irradiation or generation of photoproducts.

By employing these experimental techniques and complementary computational work (Sampedro, 2011; Fang, et al., 2018), it has been possible to unveil the photodynamics that facilitate ideal UV-filter behavior. Over the past few years, it has become clear that different families of UV-filters present different photodynamics depending on their structural properties, which are then influenced, to a greater or a lesser extent, by environmental factors (Baker and Stavros, 2016; Rodrigues and Stavros, 2018; Holt and Stavros, 2019). In particular, previous studies have found that naturally occurring UV-filters, such as cinnamates and sinapates, typically dissipate excess energy *via* ultrafast geometric photoisomerization (*E*-to-*Z* or *vice versa*). This section summarizes some of these previous findings and demonstrates how they were employed to the molecular design of improved, nature-derived UV-filters.

#### 2.4.1 Photoprotection Mechanisms in Nature

**FIGURE 7:**
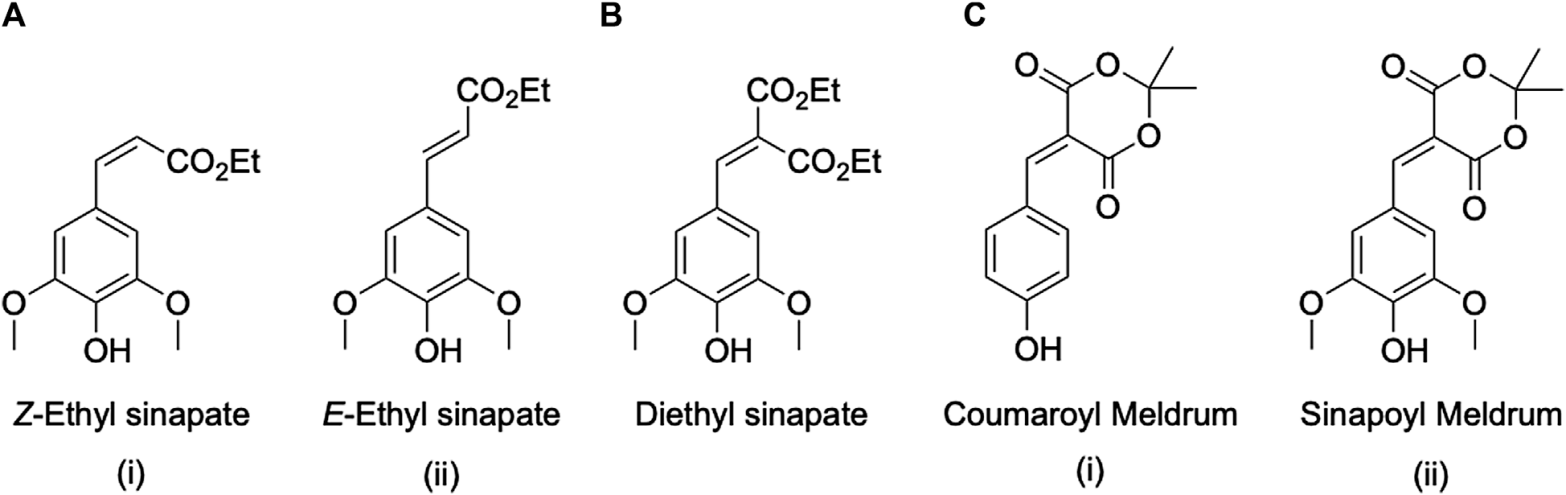
Molecular structures of the compounds studied in the case studies presented in this section, namely **(A) (i)** *Z*-Ethyl Sinapate and **(A) (ii)** *E*-Ethyl Sinapate, **(B)** Diethyl Sinapate, **(C) (i)** Coumaryl Meldrum and **(C) (ii)** Sinapoyl Meldrum.

As just discussed, geometric isomerization is one of the key non-radiative pathways by which many plant and plant-based UV-filters dissipate potentially damaging UV radiation energy into potentially harmless heat. The first case study we briefly introduce here is one of the earliest studies which provided strong evidence, through ultrafast laser spectroscopy, of *E-*to-*Z* isomerization in the model plant UV-filter ethyl sinapate, the molecular structure of which is presented in Figure 7A. In this work, Horbury et al. (2018) studied the dynamical processes in operation following absorption of UV radiation of the two isomeric forms of ethyl sinapate at their absorption peak maxima.

The isomer-specific synthesis developed by Allais and co-workers enabled Horbury et al. to study photoprotection pathways starting from either pure *E-* or *Z-*ethyl sinapate (Horbury et al., 2018). The transient absorption spectroscopy (TAS) data obtained from their fs to nanosecond (10^–9^ s, ns) TAS setup were virtually identical for both isomers. In order to elucidate the dynamical processes in operation, a sequential global fit was applied resulting in three remarkably similar time-constants (between the two isomers), which were assigned to: 1) a very fast intramolecular vibrational rearrangement, taking place within approximately 300 fs; 2) a ∼5 ps geometric photoisomerisation (*i.e. E*-to-*Z* and vice versa); and 3) a long-lived photoproduct whose presence is observed beyond the experimental time window of 2 ns. In essence, these findings suggest that UV irradiation of these sinapate species induces first a fast molecular rearrangement within solute molecules and possibly the solvent surrounding them (300 fs), followed by the photoisomerisation mechanism that allows for dissipation of most excess energy within approximately 5 ps. The photoproduct identified by Horbury *et al.* relates to the generated isomer, i.e., *E-* or *Z-*ethyl sinapate, depending on the starting isomer.

Importantly, Horbury *et al.*, were able to infer that photoisomerization was a crucial dynamical process within photoexcited ethyl sinapate by comparison of the TAS data with steady-state measurements, namely the difference spectrum resulting from subtracting the UV absorption spectrum of the pure *E* (or *Z*) ethyl sinapate from the UV absorption spectrum of the same species after 2 hours of irradiation at the molecule’s UV absorption maximum.

#### 2.4.2 Optimizing Nature’s Mechanisms

Several studies such as the one just presented have established geometric photoisomerization to be an important mechanism for energy dissipation following UV excitation of plant-based filters. However, the different isomers (e.g., *E* and *Z* isomers, see Figure 7A) often present different behaviors within a biological environment (Sharma et al., 2017), as well as having different absorption coefficients which, in practice, alters the efficacy of the UV-filter before and after radiation (Horbury et al., 2018). The key aim therefore is to synthesize a compound which prevents the formation of the alternate isomer while also maintaining the effective relaxation mechanism of *E-Z* isomerization. To this end, Horbury et al. synthesized a geometrically symmetric compound based on the sinapoyl structure, termed diethyl sinapate (Horbury et al., 2019) and shown in Figure 7B. Through the addition of two ester moieties across the acrylic bond, the formation of a geometric isomer is negated leaving only one optimized ground state structure.

Horbury et al. studied the effects of these changes on the excited state dynamics of these molecules using TAS and complementary steady-state methods. The steady-state techniques revealed diethyl sinapate to be highly photostable against solar irradiation, with only 3% of decrease in absorbance observed over a two-hour period of irradiation. The TAS which accompany these results showed that, following photoexcitation, the majority of diethyl sinapate returns to its initial, low energy form within 3 ps. Interestingly, an additional time-constant of 330 fs was required to fully capture the relaxation mechanism. This time constant was not observed in singularly substituted sinapates discussed in the previous case study of ethyl sinapate (Horbury et al., 2018). The authors attribute this time-constant to movement towards the molecular arrangement that allows energy dissipation. Furthermore, the long-lived component (>2 ns) observed in these studies is not assigned to a geometric isomer, as for the sinapates previously discussed (Horbury et al., 2018), but instead to an excited state that remains populated beyond the time-window of the experiment. Horbury *et al.* also performed TAS of diethyl sinapate on a synthetic skin solvated in alkyl benzoate, which showed the same ultrafast relaxation and therefore provide insight into the behavior of these UV-filters in an environment more comparable to a cosmetic formulation.

The findings of Horbury et al. have shown that the symmetric substitution removes concerns over potentially harmful photoproducts while also maintaining the efficient energy dissipation mechanism that is common among sinapates.

#### 2.4.3 Extrapolating to the Ideal, Synthetic and Plant-Based Sunscreen

As described, previous work identified geometric photoisomerisation as a key mechanism for energy dissipation in plant-based UV-filters, which then allowed for optimization of this mechanism to avoid significant changes in UV absorption and other properties upon photoisomerization. Building on this work, a novel class of nature-based phenolic compounds was developed with not only further optimized characteristics and photodynamics, but also absorption maxima into the UV-A range of the solar spectrum, a feature that is highly desirable in novel UV-filters (Abiola et al., 2021b). Two of these phenolic compounds, consisting of coumaryl and sinapoyl derivatives with a symmetric substitution around their acrylic bond by means of a Meldrum functional group, are shown in Figure 7C, were studied by Abiola et al. (2021b) employing ultrafast laser spectroscopy and steady-state techniques similar to those used in the studies presented above.

The studies carried out by Abiola et al. revealed that UV irradiation of these phenolic compounds leads to an initial geometry rearrangement along a charge transfer coordinate within approximately 200 fs, followed by isomerization which dissipates most excess energy within ∼450 fs (Abiola et al., 2021b). The new isomer is generated with some residual excess energy, which is dissipated as heat within 4–10 ps. The excess energy is thus almost completely dissipated within 10 ps in these phenolic compounds; there is evidence of some excess energy being trapped beyond 2 ns in the sinapoyl derivatives, but no evidence that this would lead to the generation of any potentially harmful photoproducts. These favorable ultrafast photodynamics results were also confirmed by steady-state measurements, which demonstrated that both the coumaryl and sinapoyl derivatives are highly photostable, losing less than 10% of their UV absorbance after 2 h of irradiation with a solar simulator. Furthermore, these compounds show promising antioxidant properties and no evidence of endocrine disruption effects, which makes these UV-filters suitable candidates for applications in cosmetics, for example (Peyrot et al., 2020b; Abiola et al., 2021b).

The symmetrically substituted phenolic compounds studied by Abiola et al. thus present as ideal UV-filters with strong UV-A absorption, ultrafast energy dissipation and high photostability. These results were obtained both in an industry-standard emollient and on a synthetic skin mimic, which emphasizes the suitability of the phenolic compounds under study for applications in complex environments without lessening their ideal characteristics.

The three case studies presented here demonstrate how gathering a deep understanding of UV-filter photodynamics has guided the molecular design of plant-based compounds that present ideal physico-chemical properties. This type of bottom-up approach to molecular design has proven valuable in real-life applications from photoprotective cosmetics to light-to-heat conversion materials (Abiola et al., 2021a).

## 3 Conclusion

It is undeniable that nature is just as much an inexhaustible source of elementary building blocks for (bio)chemistry as it is a source of inspiration for the design of next generation materials for wide-ranging applications. Based on this observation, the development of integrated value chains starting from biomass to analogues of natural products appears to be a promising solution for the replacement of petrochemically-sourced molecules used today. Inspired by the sinapoyl malate produced by plants, the implementation of this strategy in the field of UV-filters could only be done thanks to a multidisciplinary consortium covering fields as varied as biotechnology, chemistry, physico-chemistry and process engineering. By combining these disciplines, and through a good understanding of the mechanisms and structural features responsible for UV absorbance, we have been able to develop, optimize and integrate an unprecedented value chain that allows the production of novel sustainable UV-filters from plant-based components (i.e., carbohydrates, *p*-hydroxycinnamic acids) exhibiting impressive UV absorbance while concurrently showing equally impressive photostability. Although there are still many steps (e.g., environmental toxicity) to validate before commercializing these new UV-filters, what has been achieved so far demonstrates the strength and effectiveness of such a multidisciplinary and integrated strategy for, basically, any other type of molecule of interest.
